# *Fusobacterium nucleatum* triggers proinflammatory cell death via Z-DNA binding protein 1 in apical periodontitis

**DOI:** 10.1186/s12964-022-01005-z

**Published:** 2022-12-20

**Authors:** Hui Liu, Yuxuan Liu, Wei Fan, Bing Fan

**Affiliations:** 1 The State Key Laboratory Breeding Base of Basic Science of Stomatology (Hubei-MOST) and Key Laboratory of Oral Biomedicine Ministry of Education, School and Hospital of Stomatology, Wuhan University, 237 Luoyu Road, 430079 Wuhan, China; 2 Department of Endodontics, School and Hospital of Stomatology, Wuhan University, 237 Luoyu Road, 430079 Wuhan, China

**Keywords:** Z-DNA binding protein 1, *Fusobacterium nucleatum*, Extracellular vesicles, Cell death, Apical periodontitis

## Abstract

**Background:**

Z-DNA binding protein 1 (ZBP1) is a vital innate immune sensor that regulates inflammation during pathogen invasion. ZBP1 may contribute to pyroptosis, apoptosis and necroptosis in infectious diseases. In this study, *Fusobacterium nucleatum* (*F. nucleatum*) infection caused periapical inflammation through proinflammatory cell death and ZBP1 was involved in regulating the inflammatory activities caused by *F. nucleatum* infection in apical periodontitis (AP).

**Methods:**

Human periapical tissues were tested by fluorescent in situ hybridization, immunohistochemical staining, immunofluorescence staining, quantitative real-time PCR (qRT‒PCR) and western blotting. *F. nucleatum*-infected and *F. nucleatum* extracellular vesicles (*F. nucleatum*-EVs)-treated RAW264.7 cells were used to detect the expression of inflammatory cytokines and different cell death mechanisms by qRT‒PCR and western blotting. ZBP1 expression in *F. nucleatum*-infected tissues and RAW264.7 cells was detected by qRT‒PCR, western blotting, and immunohistochemical and immunofluorescence staining. Furthermore, the expression of ZBP1 was inhibited by siRNA and different cell death pathways, including pyroptosis, apoptosis, and necroptosis, and inflammatory cytokines were measured in *F. nucleatum*-infected RAW264.7 cells.

**Results:**

*F. nucleatum* was detected in AP tissues. *F. nucleatum*-infected RAW264.7 cells polarized to the M1 phenotype, and this was accompanied by inflammatory cytokine production. High levels of ZBP1 and GSDME (gasdermin E)-mediated pyroptosis, caspase-3-mediated apoptosis and MLKL-mediated necroptosis (PANoptosis) were identified in *F. nucleatum*-infected tissues and RAW264.7 cells. ZBP1 inhibition reduced inflammatory cytokine secretion and the occurrence of PANoptosis.

**Conclusion:**

The present study identified a previously unknown role of ZBP1 in regulating *F. nucleatum*-induced proinflammatory cell death and inflammatory activation.

**Video abstract**

**Supplementary Information:**

The online version contains supplementary material available at 10.1186/s12964-022-01005-z.

## Background

Apical periodontitis (AP) is an oral inflammatory disease with a worldwide prevalence of 52% according to a recent epidemiological study [1]. Bacterial infection in the periapical area, such as infection of *Fusobacterium nucleatum (F. nucleatum)* with its high proinflammatory ability [2, 3], is believed to be a main cause of apical inflammation. In contrast, macrophages in the periapical tissue are responsible for recognizing and eliminating bacteria. However, the process of bacterial elimination by macrophages is accompanied by complicated inflammatory activities [4, 5] and ultimately exacerbates tissue destruction. Therefore, alleviating this inflammatory activity is critical to control the progression of AP. To date, the detailed molecular mechanisms by which bacteria trigger inflammatory destruction of periapical tissues remain unclear.

Macrophage death during the antibacterial process is a major contributor to the proinflammatory response [6]. Among the most well-defined genetic pathways of cell death, pyroptosis, apoptosis and necroptosis are all involved in the regulation of host defense against pathogens and other pathologies. Pyroptosis is a more recently identified inflammatory cell death mechanism mediated by proteins belonging to the gasdermin family, and it is characterized by cell enlargement with huge bubbles and the release of abundant inflammatory substances such as interleukin 1β (IL-1β) and IL-18, following cell rupture [7, 8]. Recent studies have established that pyroptosis plays a vital function in bacterial infections [9, 10]. Mixed-lineage kinase domain-like (MLKL) proteins mediate necroptosis after being phosphorylated downstream of the receptor-interacting protein kinase 1 (RIPK1) and RIPK3 signaling axis [11]. Phosphorylated MLKL translocates to the cell membrane, resulting in cell membrane permeabilization and cell lysis [11]. By triggering the host’s innate immune response, necroptosis protects the host from pathogenic infection [12] but may also inflict tissue damage [13]. Apoptosis is thought to be a noninflammatory form of cell death, but some studies have shown that apoptosis is also accompanied by inflammatory substance production [14]. These studies imply that pyroptosis, apoptosis and necroptosis may also play a role in the defense against *F. nucleatum* infection, but additional research is required to confirm this hypothesis.

Recent studies have suggested that Z-DNA binding protein 1 (ZBP1) is an upstream receptor for pyroptosis, apoptosis and necroptosis [15]. ZBP1, also known as DNA-dependent activator of IFN regulatory factors [16], is a vital innate immune sensor that regulates inflammation during pathogen invasion. While previous research has mainly focused on the role of ZBP1 in restricting viral infection [17, 18], more recent investigations have demonstrated that ZBP1 is also involved in heatstroke [19], skin inflammation [20, 21], antitumor immunity [22] and bacterial invasion [15, 23]. Although moderate ZBP1 activation mediates host defense against certain pathogens, excessive and prolonged ZBP1 activation has been documented to paradoxically exacerbate chronic inflammation and influenza pathogenesis [20, 24]. These studies underscore the importance of a comprehensive understanding of the mechanisms mediated by ZBP1 between effective pathogen clearance and inflammation activation. However, it is unclear whether *F. nucleatum* infection activates ZBP1 and further causes proinflammatory cell death in AP.

Based on the above knowledge, the present study aimed to explore whether *F. nucleatum* infection can significantly promote periapical inflammation through proinflammatory cell death and to investigate whether and how ZBP1 is involved in regulating the inflammatory activities caused by *F. nucleatum* infection in AP.

## Materials and methods

### Ethical statement and human periapical tissue samples

The present study was approved by the Ethics Committee of School and Hospital of Stomatology, Wuhan University, in accordance with the institutional guidelines (2022LUNSHENZIB16) and followed the Declaration of Helsinki of the World Medical Association [25]. All patients agreed to participate in this study. Tissue samples were obtained from 28 patients, including 10 normal tissues and 18 AP tissues. The normal periapical tissues consisted of third molars extracted for orthodontic treatment, without any caries lesions or periodontitis. The AP tissues consisted of teeth collected from patients diagnosed with chronic AP who needed apical surgery or extraction.

### Cell culture

RAW264.7 cells (ATCC) were grown in DMEM (GIBCO) supplemented with 10% FBS (GIBCO). Cells (1 × 10^6^ cells/well into 6-well plates, or 5 × 10^5^ cells/well into 12-well plates) were seeded and incubated overnight before usage. The cells were incubated with lipopolysaccharide (LPS, 100 ng/ml, Sigma) or recombinant murine interleukin-4 (IL-4, 20 ng/ml, Peprotech) for 24 h to induce M1 macrophage or M2 macrophage polarization, respectively [26].

### Quantitative real-time PCR (qRT‒PCR)

Total RNA from tissues or in vitro cultured cells was extracted with TRIzol reagent (Life Technologies) and cDNA was prepared with a Hiscript II Q RT SuperMix cDNA synthesis kit (Vazyme Biotech, China). qRT**‒**PCR was performed to detect relative mRNA expression levels using SYBR Green master mix (Vazyme Biotech, China) on a QuantStudio 6 Real-Time PCR System (Life Technologies). The 2^−ΔΔCT^ method was used to quantify fold induction. Each cDNA data point was normalized to GAPDH expression. The primer sequences are shown in Additional file 1: Table S1.

### siRNA-mediated gene silencing

siRNAs against mouse ZBP1, RIPK3, GSDME and MLKL were purchased from GenePharma (Shanghai, China) and transfected using RNAi Lipofectamine (Invitrogen) following the manufacturer’s instructions. Nontargeting control siRNA was used as a negative control, and qRT‒PCR was performed to determine the knockdown efficiency. The siRNA sequences are shown in Additional file 1: Table S2.

### Western blot analysis

Tissues or in vitro cultured cells were lysed in RIPA buffer (Beyotime, China) containing protease and phosphatase inhibitors (Roche). Western blotting was performed according to the following procedure [27]. Proteins were boiled and separated by electrophoresis through 12% SDS‒PAGE gels. Following electrophoretic transfer of proteins onto polyvinylidene difluoride membranes (Roche), nonspecific binding was blocked by incubation with 5% skimmed milk. The membranes were incubated at 4 °C overnight with the following primary antibodies: anti-ZBP1 (AG-20B-0010, AdipoGen, 1:1000); anti-GSDME (ab215191, Abcam, 1:1000); anti-cleaved-caspase-3 (#9664, Cell Signaling Technology, 1:1000); anti-pMLKL (ab196436, Abcam, 1:1000); anti-pMLKL (TA7420S, Abmart, 1:1000); and anti-β-Actin (PMK058, BIOPRIMACY, 1:1000). The membranes were then washed and incubated with secondary antibodies (1:5000, Proteintech, China). An ECL kit was used (Advantista) to detect the protein bands, and the Odyssey system (LI-COR Biosciences) was used for visualization. Images were analyzed with ImageJ software (ImageJ, National Institutes of Health, Bethesda, Maryland, USA, https://imagej.nih.gov/ij/, 1997–2018).

### Bacterial culture and cell infection

*F. nucleatum* (ATCC 25586) was grown in brain heart infusion (BHI) broth (BD) supplemented with hemin and vitamin K1 or on Columbia agar plates (LAND BRIDGE, China). The bacteria were grown in an anaerobic chamber (Mart Microbiology, Netherlands) in an atmosphere of 90% N_2_, 5% CO_2_, and 5% H_2_ at 37 °C. For *F. nucleatum* infection, cells were infected in DMEM supplemented with 10% FBS at an MOI of 1, 10, 20, 50 or 100 (12 h) to determine the secretion of inflammatory cytokines, and at an MOI of 50 (0, 6, 12, 24 or 48 h) to determine the gene expression of *Gsdme*, *Caspase-3* and *Mlkl* as well as the protein expression of N-GSDME, cleaved-caspase 3 and pMLKL. For live/dead staining, an MOI of 100 (0, 6, 12, 24 and 48 h) was used. To verify the phagocytosis of macrophages, *F. nucleatum* was stained with CFDA SE (5 mM, Beyotime, China).

### Live/dead staining

RAW264.7 cells were seeded into 12-well plates (5 × 10^5^ cells/well) and infected with *F. nucleatum*. After incubation for 0, 6, 12, 24 and 48 h, 100 nM PI (Thermo Fisher Scientific) and 1 µM calcein AM (Beyotime, Shanghai, China) were added to the cells [28]. Images were acquired on a Zeiss LSM880 Fast microscope using ZEN Software (Zeiss).

### Hematoxylin and eosin (HE) and immunohistochemistry (IHC) staining

Tissues were fixed in 4% paraformaldehyde, embedded in paraffin, and cut into 4 μm sections. IHC was performed according to a previously reported procedure [27]. In brief, sections were dewaxed in xylene and rehydrated in gradient alcohol, and citrate buffer at pH 6.0 was used for heat-induced antigen retrieval. For HE staining, sections were stained for 2 min in hematoxylin, differentiated in tap water (15 min) and incubated for 1 min in eosin. For IHC, sections were incubated in endogenous peroxidase blocking agent and goat serum at 37 °C for 20 min. Then, primary antibodies (anti-ZBP1, AG-20B-0010, AdipoGen, 1:200 and anti-CD68, Cell Signaling Technology, #26042, 1:200) were added to the sectioned tissues, and they were incubated overnight at 4 °C. A biotinylated secondary antibody and the anti-biotin-peroxidase reagent were added to the sections, and they were incubated at 37 °C for 20 min. DAB substrate (Mxb Biotechnologies) was used to visualize the staining. All samples were incubated for the same amount of time with DAB substrate, and the nuclei were stained with hematoxylin. All slides were scanned by an Aperio ScanScope CS scanner (Aperio).

### Immunofluorescence staining

Immunofluorescence staining was performed on cells or tissue sections as previously described [29]. Cells were plated in 12-well plates, allowed to adhere overnight and infected with CFDA SE-labeled *F. nucleatum* at an MOI of 50. Following infection, cells were fixed in 4% paraformaldehyde, permeabilized in 0.2% Triton X-100 and blocked with 5% bovine serum albumin for 1 h. For sections, antigen retrieval and endogenous peroxidase blocking were performed according to the description in IHC. The cells or sections were incubated with primary antibodies (anti-CD68, CST, #26042, 1:200; anti-ZBP1, AG-20B-0010, AdipoGen, 1:200; anti-Z-DNA, absolute antibody, Ab00783-3.0, 1:200) overnight at 4 ºC, followed by incubation with fluorophore-conjugated secondary antibodies (Abbkine) for 1 h. Nuclei were stained with DAPI (Beyotime, China). Images were acquired on a Zeiss LSM880 Fast microscope using ZEN Software (Zeiss).

### Fluorescent in situ hybridization (FISH)

FISH of *F. nucleatum* on formalin-fixed paraffin-embedded tissues was performed manually following the manufacturer’s instructions. Briefly, the slides were dewaxed in xylene and rehydrated in gradient alcohol, and citrate buffer at pH 6.0 was used for heat-induced antigen retrieval. The slides were incubated with protease K solution (20 µg/ml) at 37 °C for 30 min and then subjected to hybridization buffer at 37 °C for 1 h. The hybridization buffer was removed and incubated with hybridization buffer containing the *F. nucleatum* probe (10 ng/µl) overnight at 37 °C. Then, the tissues were washed with saline sodium citrate buffer and incubated with DAPI for 30 min at room temperature. Images were acquired on a Zeiss LSM880 Fast microscope using ZEN Software (Zeiss).

### Preparation and characterization of *F. nucleatum* extracellular vesicles (*F. nucleatum*-EVs)

For the isolation of *F. nucleatum*-EVs, *F. nucleatum*-EVs were pelleted by sequential centrifugation at 3000 × g for 30 min and 15,000 × g for 30 min at 4 °C. The supernatant was filtered by a 0.22 μm filter (Millipore) and then centrifuged for 3 h at 150,000 g and 4 °C. The obtained *F. nucleatum*-EVs were resuspended in PBS. Transmission electron microscopy (TEM; JEM-2100, JEOL, Tokyo, Japan) and dynamic light scattering (DLS; Malvern, Zetasizer Nano ZS, UK) were used to assess the morphologies and diameters of *F. nucleatum*-EVs. Zeta potential was measured by a zeta potential analyzer (Malvern, Zetasizer Nano ZS, UK). Dio-labeled *F. nucleatum*-EVs were cultured with RAW 264.7 cells to verify the internalization of *F. nucleatum*-EVs.

### Apoptosis analysis

Apoptosis was detected by an annexin V-FITC/PI apoptosis kit (Chamot Biotechnologies, Shanghai, China). RAW264.7 cells were cultured with *F. nucleatum*-EVs at a dose of 0–5 µg/ml. Annexin V-FITC was detected through the FITC detection channel, and PI was detected through the PI detection channel on a Beckman CytoFLEX Flow Cytometer (Beckman Coulter, Brea, CA, USA). For this experiment, 10 000 events were analyzed for each sample.

### Statistical analysis

GraphPad Prism 8.0 was used for data analysis. Student’s *t* test and one-way ANOVA tests (multiple groups) were used for comparison. Data are shown as the mean ± SEM. P < 0.05 was considered statistically significant (*P < 0.05; **P < 0.01; ***P < 0.001).

## Results

### Recruitment of macrophages and inflammatory activation in *F. nucleatum*-infected AP tissues

**Fig. 1:**
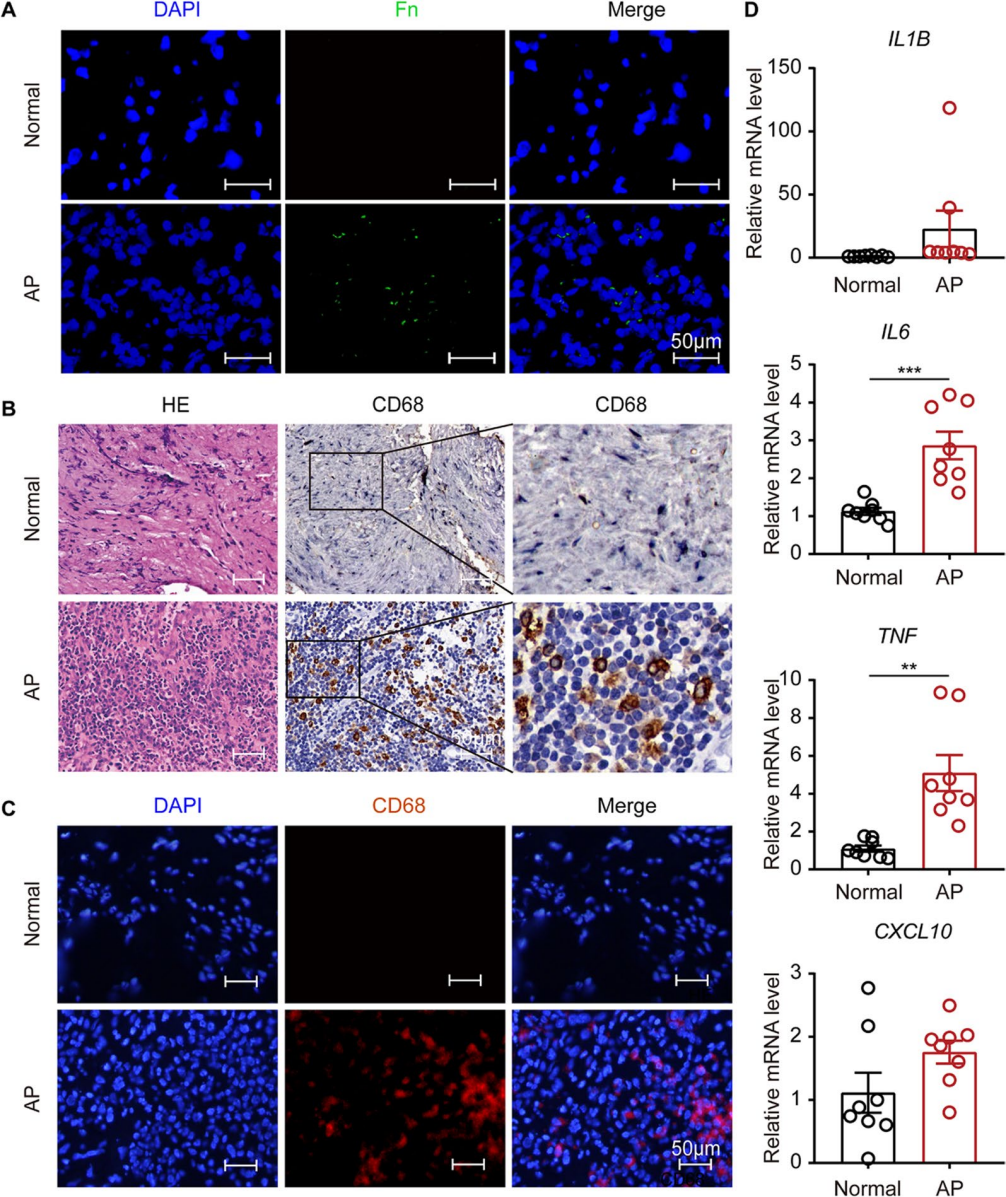
Recruitment of macrophages and production of inflammatory cytokines in *F. nucleatum*-infected AP tissues. **A** Representative image of FISH in periapical tissues (AP, n = 18; Normal, n = 10; Scale bar, 50 μm). **B** Immunohistochemical staining of CD68 in AP or normal tissues. (AP, n = 10; Normal, n = 10; Scale bar, 50 μm). **C** Immunofluorescence staining of CD68 (red) in AP or normal tissues. (AP, n = 10; Normal, n = 10; Scale bar, 50 μm). **D** mRNA levels of *IL1B*, *IL6*, *TNF* and *CXCL10* detected by qRT-PCR. (AP, n = 8; Normal, n = 8). Data are expressed as the mean ± SEM (unpaired *t* test; *p < 0.05; **p < 0.01; ***p < 0.001)

Human periapical tissues were analyzed by FISH to examine the presence of *F. nucleatum* in AP tissues. The results showed that *F. nucleatum* was present in 10 samples out of 18 AP samples, but was not detected in normal samples (Fig. 1A). To investigate whether the innate immune system is activated in *F. nucleatum*-infected tissues, the 10 AP samples infected with *F. nucleatum* and 10 normal samples were analyzed by immunohistochemistry and immunofluorescence. The results showed that CD68^+^ macrophage recruitment was enhanced in *F. nucleatum*-infected samples compared to normal samples (Fig. 1B, C). Additionally, the expression levels of inflammatory cytokines (*IL1B, IL6, TNF* and *CXCL10*) were higher in *F. nucleatum*-infected AP samples than in normal tissues (Fig. 1D).

### *F. nucleatum* infection increases M1 phenotype macrophages and the production of inflammatory cytokines in vitro

**Fig. 2:**
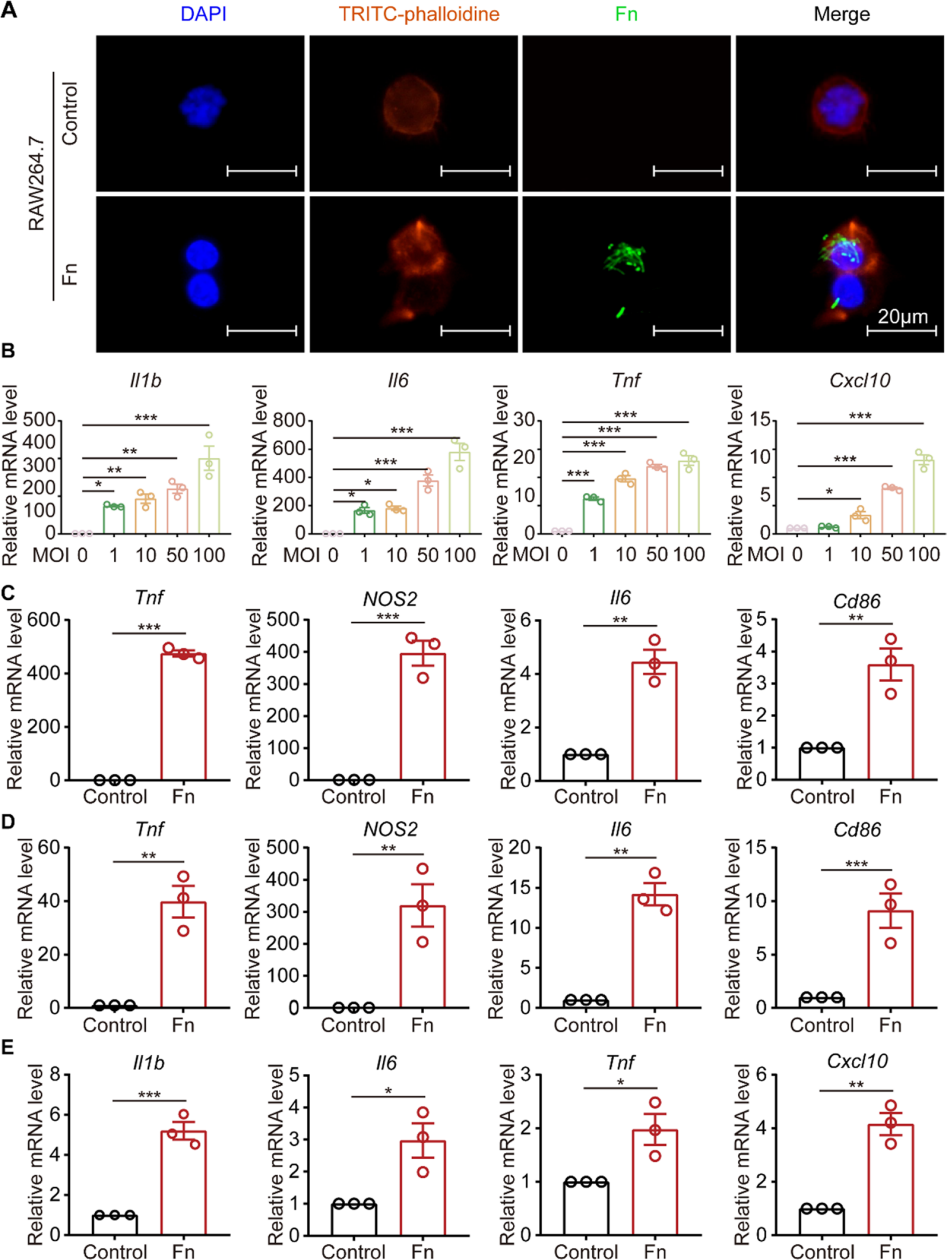
* F. nucleatum* increases M1 macrophages and inflammatory cytokines production. **A** Representative image of *F. nucleatum* staining in normal and *F. nucleatum*-treated cells (Scale bar, 20 μm). **B** mRNA levels of *Il1b*, *Il6*, *Tnf* and *Cxcl10* in RAW264.7 cells cocultured with *F. nucleatum* (MOI = 0, 1, 10, 50 and 100) for 12 h (one-way ANOVA). **(C)** mRNA levels of *Tnf*, *Nos2*, *Il6* and *Cd86* in M2 RAW264.7 cells cocultured with *F. nucleatum* (MOI = 50) for 12 h (unpaired *t* test). **D** mRNA levels of *Tnf*, *Nos2*, *Il6* and *Cd86* in M0 RAW264.7 cells cocultured with *F. nucleatum* (MOI = 50) for 12 h (unpaired *t* test). **E** mRNA levels of *Il1b*, *Il6*, *Tnf* and *Cxcl10* in M1 RAW264.7 cells cocultured with *F. nucleatum* (MOI = 50) for 12 h (unpaired *t* test). Data are expressed as the mean ± SEM (*p < 0.05; **p < 0.01; ***p < 0.001)

To explore the phagocytosis of macrophages during *F. nucleatum* infection, CFDA SE labeled *F. nucleatum* was cocultured with macrophages. The results showed that macrophages accumulated a considerable quantity of fluorescence-labeled *F. nucleatum* (Fig. 2A). To further discern the effect of *F. nucleatum* infection on the macrophage phenotype and the production of inflammatory cytokines, macrophages of the M1 (typical markers are shown in Additional file 1: Fig. S1A) or M2 phenotype (typical markers are shown in Additional file 1: Fig. S1B) were induced in vitro. *F. nucleatum* was then cocultured with M0, M1 and M2 macrophages. The qRT‒PCR results revealed that both M0 and M2 macrophages began to polarize to the M1 phenotype when cocultured with *F. nucleatum* (Fig. 2 C, D), and M1 macrophages were induced to secrete inflammatory factors after coculture with *F. nucleatum* (Fig. 2E). Furthermore, the qRT‒PCR results showed that cytokines (*Il1b, Il6, Tnf* and *Cxcl10*) were significantly upregulated by *F. nucleatum* infection in a dose-dependent manner (Fig. 2B).

### *F. nucleatum* infection triggers the production of inflammatory cytokines through activation of ZBP1

**Fig. 3:**
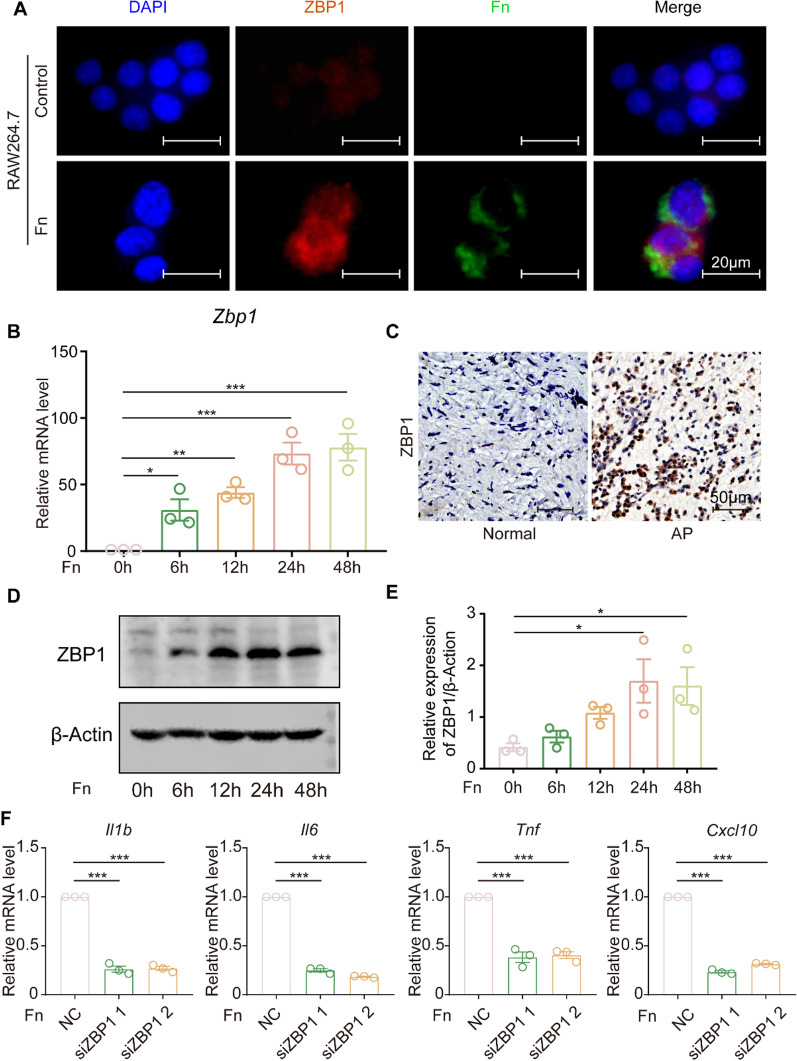
* F. nucleatum* triggers the inflammatory cytokines production through the activation of ZBP1. **A** Immunofluorescence staining of ZBP1 in normal and *F. nucleatum*-treated cells (Scale bar, 20 μm). **B**–**E** mRNA and protein levels of ZBP1 in RAW264.7 cells cocultured with *F. nucleatum* (MOI = 50) for 0, 6, 12, 24 and 48 h. The quantitative data represent the relative ratio of the target protein to β-Actin (one-way ANOVA). **C** Immunohistochemistry staining of ZBP1 in normal and AP tissues (AP, n = 10; Normal, n = 10; Scale bar, 50 μm). **F** mRNA levels of *Il1b*, *Il6*, *Tnf* and *Cxcl10* in RAW264.7 cells cocultured with *F. nucleatum* (MOI = 50) for 12 h after ZBP1 silencing (NC, negative control; one-way ANOVA). Data are expressed as the mean ± SEM (*p < 0.05; **p < 0.01; ***p < 0.001)

To elucidate how *F. nucleatum* causes inflammation in macrophages, qRT‒PCR was performed, and the results demonstrated that bacterial infection activated ZBP1 mRNA expression in macrophages in vitro (Fig. 3B). ZBP1 protein expression was further studied following *F. nucleatum* infection. ZBP1 expression was increased in a time-dependent manner, with a greater increase at 24 and 48 h than at 6 and 12 h post-infection (Fig. 3D, E). Consistently, immunofluorescence showed high ZBP1 protein expression following *F. nucleatum* infection (Fig. 3A). Further analysis of ZBP1 expression in tissues revealed that it was expressed at a higher level in infected tissues than in normal tissues (Fig. 3C and Additional file 1: Fig. S2A). ZBP1 expression was then suppressed with siRNA (Additional file 1: Fig. S2B), and the results showed that the production of cytokines (*Il1b*, *Il6*, *Tnf* and *Cxcl10*) in AP tissues was significantly decreased (Fig. 3F).

### Sustained ZBP1 activation induces pyroptosis, apoptosis and necroptosis in macrophages

**Fig. 4:**
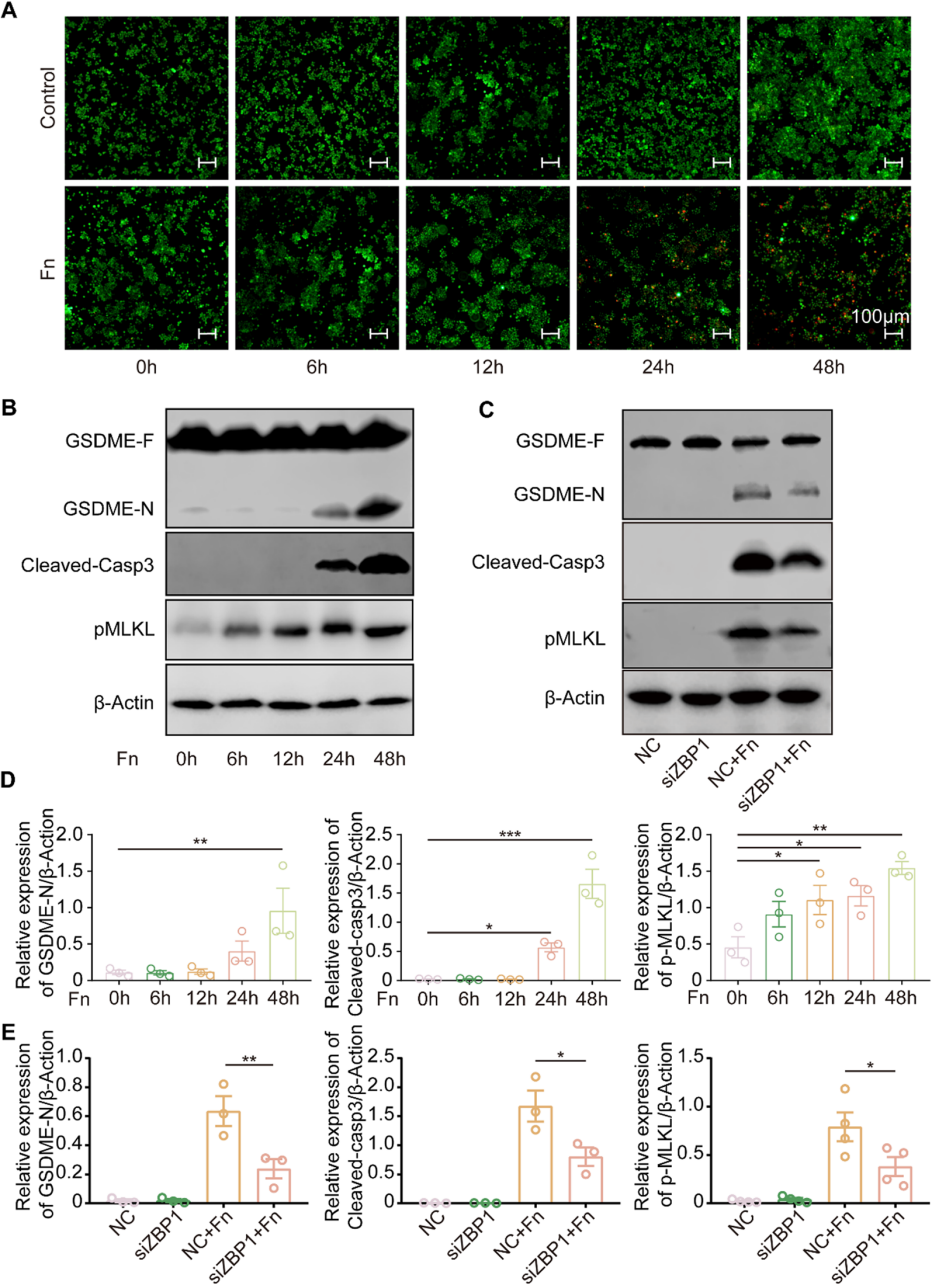
Sustained ZBP1 activation induces pyroptosis, apoptosis and necroptosis in macrophages. **A** Live/dead staining of RAW264.7 cells at 0, 6, 12, 24 and 48 h post-*F. nucleatum* infection (MOI = 100, live cells (green); dead cells (red); Scale bar, 100 μm). **B**,** D** Protein levels of N-GSDME, cleaved-caspase-3 and pMLKL in RAW264.7 cells cocultured with *F. nucleatum* (MOI = 50) for 0, 6, 12, 24 and 48 h. The quantitative data represent the relative ratio of the target protein to β-Actin (one-way ANOVA). **C**,** E** Protein levels of N-GSDME, cleaved-caspase-3 and pMLKL in RAW264.7 cells cocultured with *F. nucleatum* (MOI = 50) after ZBP1 knockdown (NC, negative control). Data are expressed as the mean ± SEM (*p < 0.05; **p < 0.01; ***p < 0.001)

**Fig. 5:**
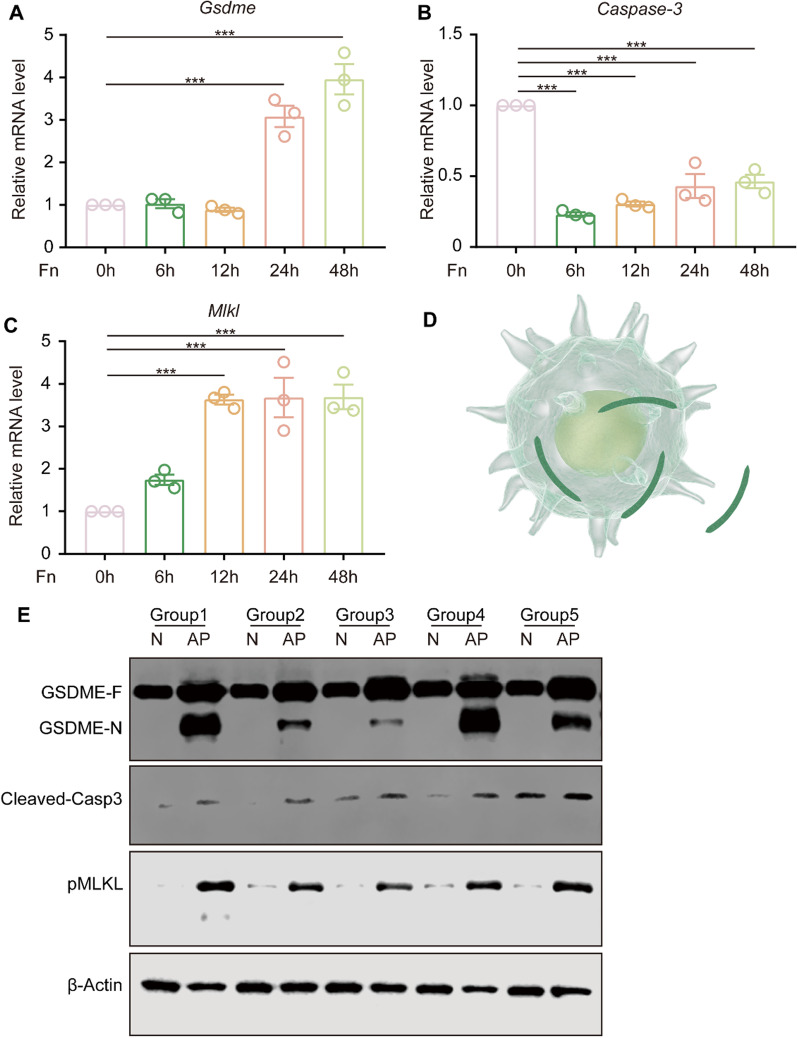
*Gsdme*, *Caspase-3*, and *Mlkl* expression in RAW264.7 cells and periapical tissues. **A-C** mRNA levels of *Gsdme*, *Caspase-3* and *Mlkl* in RAW264.7 cells cocultured with *F. nucleatum* (MOI = 50) for 0, 6, 12, 24 and 48 h (one-way ANOVA). **D** Macrophages infected with *F. nucleatum*. **E** Protein levels of N-GSDME, cleaved-caspase-3 and pMLKL in normal tissues and AP tissues (AP, n = 5; Normal, n = 5). Data are expressed as the mean ± SEM (***p < 0.001)

Live/dead staining was utilized to determine whether persistent *F. nucleatum* infection results in macrophage death. The results indicated that macrophages began to exhibit death at 24 h post-infection, and more pronounced death at 48 h post-infection (Fig. 4 A). Further investigation was performed by western blot analysis and revealed a time-dependent increase in GSDME cleavage, cleaved-caspase-3 and pMLKL (Fig. 4B, D), indicating that *F. nucleatum* infection induced a mixed mechanism of cell death pathways, including pyroptosis, apoptosis and necroptosis. qRT‒PCR analysis revealed that the expression of *Gsdme* and *Mlkl* was increased after infection, while the expression of *caspase-3* was decreased (Fig. 5A, B, C). In accordance with those results, using periapical tissue samples, the expression levels of N-GSDME, cleaved-caspase-3 and pMLKL were significantly increased in AP tissue compared to normal samples (Fig. 5E). ZBP1 knockdown significantly reduced the expression of N-GSDME, cleaved-caspase 3 and pMLKL after *F. nucleatum* infection (Fig. 4C, E).

### Effects of different cell death pathways on the production of inflammatory cytokines

**Fig. 6:**
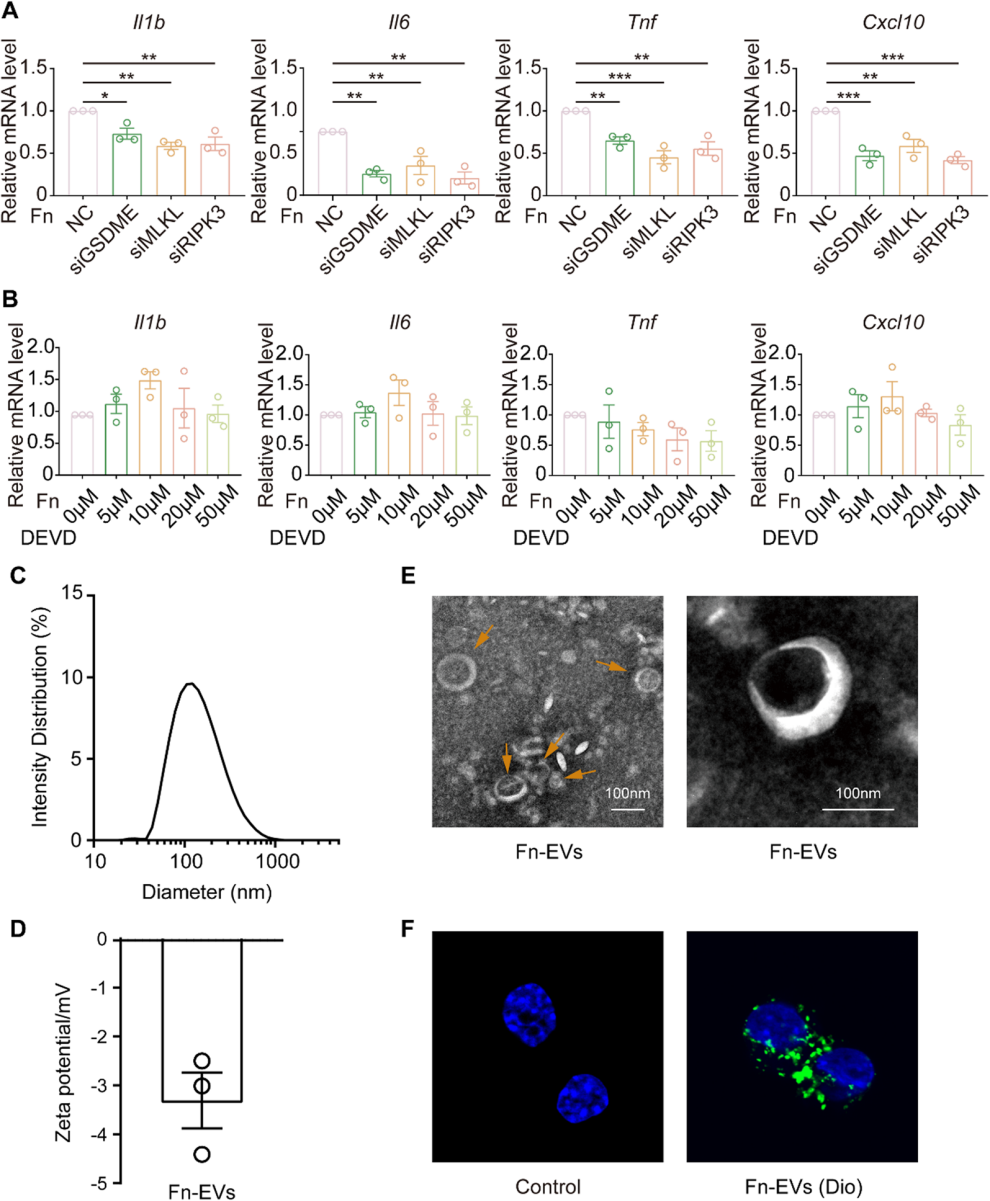
Inhibition of pyroptosis and necroptosis reduces the production of inflammatory cytokines. **A** mRNA levels of *Il1b*, *Il6*, *Tnf* and *Cxcl10* in RAW264.7 cells cocultured with *F. nucleatum* (MOI = 50) for 12 h after transfection with siGSDME, siMLKL or siRIPK3 for 48 h (NC, negative control; one-way ANOVA). **B** mRNA levels of *Il1b*, *Il6*, *Tnf* and *Cxcl10* in RAW264.7 cells cocultured with *F. nucleatum* (MOI = 50) for 12 h after treatment with the DEVD caspase-3 inhibitor at 5, 10, 20, and 50 µM (one-way ANOVA). **C, E** Diameter distribution and morphology of *F. nucleatum*-EVs. **D** Zeta potential of *F. nucleatum*-EVs. **F** Images of nuclei of RAW 264.7 and Dio-labeled *F. nucleatum*-EVs (green) after internalization by RAW 264.7 cells. Data are expressed as the mean ± SEM (*p < 0.05; **p < 0.01; ***p < 0.001)

To further elucidate the involvement of different cell death pathways in inflammation, inflammatory cytokines were detected after the inhibition of cell death including pyroptosis, apoptosis and necroptosis. GSDME expression was knocked down using siRNA, which resulted in decreased inflammatory cytokine (*Il1b, Il6, Tnf* and *Cxcl10*) release (Fig. 6A). Functional knockdown of RIPK3 and MLKL with siRNA resulted in a significant decrease in the release of inflammatory cytokines (*Il1b, Il6, Tnf* and *Cxcl10*) (Fig. 6A). However, when caspase-3 was blocked using caspase-3 inhibitors, the release of inflammatory factors (*Il1b, Il6, Tnf* and *Cxcl10*) did not significantly decrease (Fig. 6B).

### Effects of *F. nucleatum*-EVs on inflammation cytokine production and cell death

**Fig. 7:**
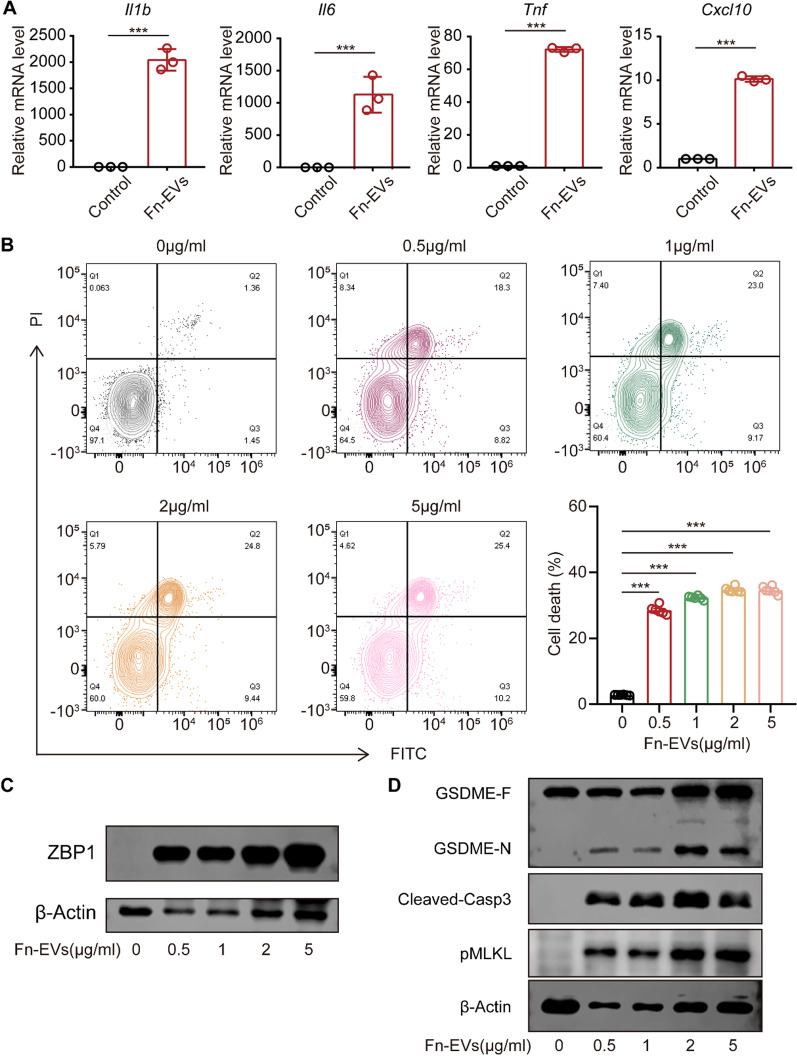
* F. nucleatum*-EVs induced pyroptosis, apoptosis and necroptosis in macrophages. **A** mRNA levels of *Il1b*, *Il6*, *Tnf* and *Cxcl10* in RAW264.7 cells cocultured with *F. nucleatum* (1 µg/ml) for 12 h. **B** Cell death after cotreatment with *F. nucleatum*-EVs at the dose of 0 to 5 µg/ml. **C** Protein levels of ZBP1 in RAW264.7 cells cocultured with *F. nucleatum*-EVs (0–5 µg/ml). **D** Protein levels of N-GSDME, cleaved-caspase-3 and pMLKL in RAW264.7 cells cocultured with *F. nucleatum*-EVs (0–5 µg/ml). Data are expressed as the mean ± SEM (*p < 0.05; **p < 0.01; ***p < 0.001)

The DLS (Fig. 6 C) and TEM (Fig. 6E) results verified isolation of *F. nucleatum*-EVs from the culture medium of *F. nucleatum*, and zeta potential is shown in Fig. 6D. The Dio-labeled *F. nucleatum*-EVs accumulated in RAW 264.7 cells (Fig. 6F). qRT‒PCR results demonstrated that the production of inflammatory factors by RAW 264.7 cells increased significantly after culture with *F. nucleatum*-EVs (Fig. 7A). In addition, cotreatment with *F. nucleatum*-EVs resulted in an increase in RAW264.7 cells death (Fig. 7B). Furthermore, the results of western blot analysis revealed an increased expression of ZBP1 and Z-nucleic acid (Fig. 7C and Additional file 1: Fig. S4), cleaved GSDME, cleaved-caspase-3 and pMLKL (Fig. 7D), indicating that *F. nucleatum* infection induced a mixed mechanism of cell death pathways, including pyroptosis, apoptosis and necroptosis.

## Discussion

**Fig. 8:**
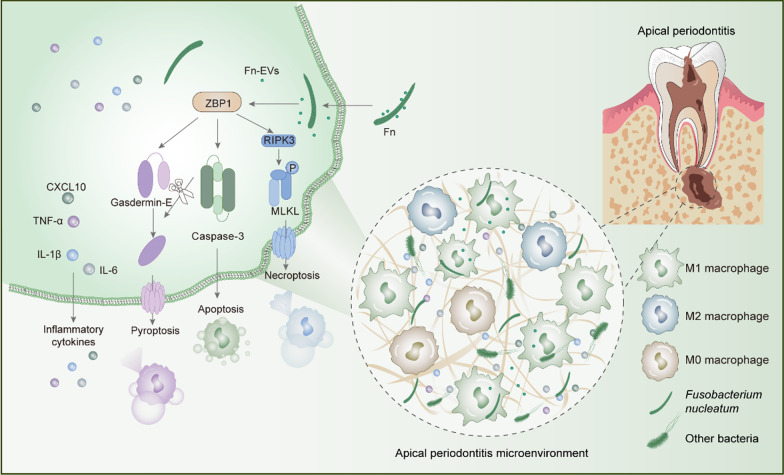
Mechanism illustration of *F. nucleatum*-induced inflammation in AP. *F. nucleatum* infection triggers the activation of ZBP1 by releasing EVs in macrophages. On the one hand, activated ZBP1 promotes the cleavage of GSDME, thereby liberating N-GSDME to induce pyroptosis. On the other hand, activated ZBP1 activates RIPK3, which then promotes the phosphorylation of MLKL to induce necroptosis. Both pyroptotic and necroptotic pathways result in lytic cell death, thereby leading to the release of inflammatory cytokines

Previous studies have identified the role of numerous bacteria in the occurrence of AP [30–32]. However, the intricate proinflammatory mechanisms of bacteria in the pathogenesis of AP are still unclear. Recent findings have demonstrated ZBP1 upregulation during pathogen invasion [15], suggesting that it may contribute to mediation of the inflammatory response. The present study first found that *F. nucleatum* activated ZBP1, and further examination revealed the connection between cell death and ZBP1 (Fig. 8). Thus, the present study tried to elucidate the mechanism by which *F. nucleatum* exacerbates periapical inflammation via ZBP1 activation.

Macrophages are present in the infected pulp and periapical tissues, and they perform a critical protective function against persistent periapical infection [33, 34]. The present study discovered significant macrophage infiltration in the infected periapical tissue. In addition, *F. nucleatum* and considerable accumulation of inflammatory cytokines were observed in AP tissues. These findings demonstrated that infection with *F. nucleatum* enhanced macrophage recruitment and was positively correlated with inflammation in AP tissues. To further confirm the link between *F. nucleatum* infection and inflammation, macrophages were cocultured with *F. nucleatum* in vitro. The results indicated that macrophages perform extensive phagocytosis to remove invading *F. nucleatum*, a process that is accompanied by macrophage polarization to the M1 phenotype and the production of inflammatory cytokines. Consistent with previous studies, macrophages are actively involved in the antimicrobial process, but the associated inflammatory response during this process leads to damage and serious disease [35, 36].

Programmed cell death of macrophages is well integrated into antibacterial immune responses during infection and is critical for the elimination of bacteria [37–40]. According to the present experimental results, macrophage death increased gradually with infection development, and significant cell death occurred 48 h after infection. To elucidate the mechanisms underlying this phenomenon, the presence of different types of programmed cell death was further examined. The results showed that *F. nucleatum* infection ultimately caused pyroptosis, apoptosis and necroptosis. In accordance with this finding, *F. nucleatum*-EVs induced significant cell death after being cocultured with RAW264.7 cells.

Pyroptosis and necroptosis have been defined as proinflammatory cell death processes that are characterized by the release of cytokines and activation of the immune system [41, 42]. GSDME belongs to the gasdermin family and functions as an executor of pyroptosis. It contains N- and C-terminal domains, and the N-terminal monomers oligomerize to form pores in the plasma membrane [43]. Recent studies have suggested that GSDME promotes the release of inflammatory cytokines [44, 45]. Consistently, the findings of this study indicated that activation of ZBP1 further promoted cleavage of GSDME and MLKL phosphorylation, resulting in cell lysis and release of inflammatory substances. While apoptosis is historically considered immunologically silent, studies of the crosstalk between apoptosis and pyroptosis have indicated that in some cell types, apoptotic cell death is also inflammatory [37, 46]. The present results showed that functional inhibition of apoptosis had little effect on inflammation. These findings may indicate that pyroptosis and necroptosis, rather than apoptosis, play a significant role in periapical inflammation. Accumulating evidence suggests that different death processes are not mutually exclusive but interact extensively. As a well-characterized apoptotic executor, caspase-3 also induces GSDME cleavage [46]. Although inhibition of GSDME decreased inflammation in the present study, inhibition of caspase-3 had no effect on inflammation, which may imply the existence of other compensatory mechanisms and highlights the complexity of AP. For example, RIPK3 has been implicated in both apoptosis and necroptosis signaling, and it has also been discovered that RIPK3 causes NLRP3 activation to mediate pyroptosis [47]. The present results indicate that restriction of pyroptosis and necroptosis as well as enhanced apoptosis may clear pathogens without inducing considerable inflammation. The intricacy of various types of cell death demonstrates the necessity of investigating regulatory mechanisms. The finding of ZBP1 activation in response to *F. nucleatum* infection offered insights into the mechanism underlying *F. nucleatum*-induced pyroptosis, apoptosis and necroptosis.

ZBP1 is recognized as an immune sensor regulating the activation of both programmed cell death and inflammation under diverse conditions including pathogen invasion and embryonic development. The most common role of ZBP1 is its involvement in antiviral responses [48], but in addition to regulating virus invasion, ZBP1 may also be required for bacterial infection, such as Yersinia pestis [23] and Francisella infection [49]. In this study, ZBP1 was highly expressed in AP tissues, and *F. nucleatum* infection stimulated ZBP1 expression in vitro. Inhibiting ZBP1 expression resulted in a significant reduction in inflammatory cytokines. These observations suggest that *F. nucleatum* triggers an inflammatory response in AP by activating ZBP1. Recent studies have demonstrated that ZBP1 activation can induce programmed cell death. For example, ZBP1 induces necroptosis via an RHIM-mediated homotypic interaction with RIPK3 [48, 50], and pyroptosis is also activated upon ZBP1 activation [51, 52]. Consistently, the present results showed that inhibition of ZBP1 reduced pyroptosis, apoptosis and necroptosis of macrophages during *F. nucleatum* infection. ZBP1 was initially recognized as a sensor for double-stranded DNA (dsDNA), and further research demonstrated its role as a Z-nucleic acid sensor to identify both endogenous and viral Z-nucleic acid [17, 53]. Therefore, it is hypothesized that Z-nucleic acid was present in RAW264.7 cells stimulated with *F. nucleatum*-EVs, which then led to the activation of ZBP1. Our findings confirmed that with the stimulation of *F. nucleatum*-EVs, Z-nucleic acid was detected and consistent with the expression level of ZBP1 protein (Additional file 1: Fig. S4). Based on this finding, it might be surmised that Z-nucleic acid during *F. nucleatum* infection is a molecular mechanism that activates the upregulation of ZBP1 in AP although more studies are needed to verify this role.

## Conclusion

The present study identified a previously unknown role of ZBP1 in regulating *F. nucleatum*-induced proinflammatory cell death and inflammation activation, and targeting ZBP1 may prevent serious periapical damage. The in vivo role of ZBP1 during AP development still requires further investigation.

## Supplementary Information

**Additional file 1**. **Table S1**. qRT-PCR primers used in the present study. **Table S2**. siRNA sequences used in the present study. **Fig. S1**. M0 macrophage differentiated into M1 and M2 phenotype. **A** mRNA levels of Tnf, Nos2, Il6 and Cd86 in RAW264.7 cells treated with LPS (100 ng/ml) for 24 h (unpaired t test). **B** mRNA levels of Tgfb, Arg1, Il10 and Cd206 in RAW264.7 cells treated with IL-4 (20 ng/ml) for 24 h (unpaired t test). Data are expressed as the mean ± SEM (*p < 0.05; **p < 0.01; ***p < 0.001). **Fig. S2**. Enhanced ZBP1 expression in *F. nucleatum* infected AP tissues and knockdown efficiency of ZBP1. **A** Immunohistochemistry staining of ZBP1 in the normal and AP tissues (AP, n = 10; Normal, n = 10; Scale bar, 50 μm). **B** siRNA knockdown efficiency of ZBP1 in RAW264.7 cells (NC, negative control). **Fig. S3**. siRNA knockdown efficiency of GSDME, MLKL and RIPK3 in RAW264.7 cells. **A** siRNA knockdown efficiency of GSDME, MLKL and RIPK3 in RAW264.7 cells (NC, negative control). **Fig. S4** Immunofluorescence staining of Z-nucleic acid and ZBP1 in RAW264.7 cells treated with Fn-EVs (5 µg/ml) for 12 h (Scale bar, 20 μm).

## Data Availability

The data used to support the findings of this study are included within the article and the supplementary information file.

## Abbreviations

AP: Apical periodontitis
FISH: Fluorescent in situ hybridization
ZBP1: Z-DNA binding protein 1
*F. nucleatum*: *Fusobacterium nucleatum*
EVs: Extracellular vesicles
MLKL: Mixed-lineage kinase domain-like
LPS: Lipopolysaccharide
IL-4: Interleukin-4
RIPK1: RIP like protein kinase 1
RIPK3: RIP like protein kinase 3
GSDME: Gasdermin E
TEM: Transmission electron microscope
DLS: Dynamic light scattering

## References

[1] Tiburcio-Machado CS, Michelon C, Zanatta FB, Gomes MS, Marin JA, Bier CA (2021). The global prevalence of apical periodontitis: a systematic review and meta-analysis. Int Endod J.

[2] Amaral RR, Braga T, Siqueira JF, Rocas IN, da Costa Rachid CTC, Oliveira AGG, de Souza Cortes MI, Love RM (2022). Root Canal Microbiome Associated with asymptomatic apical periodontitis as determined by high-throughput sequencing. J Endod.

[3] Chow AT, Quah SY, Bergenholtz G, Lim KC, Yu VSH, Tan KS (2019). Bacterial species associated with persistent apical periodontitis exert differential effects on osteogenic differentiation. Int Endod J.

[4] Simpson DS, Pang J, Weir A, Kong IY, Fritsch M, Rashidi M, Cooney JP, Davidson KC, Speir M, Djajawi TM (2022). Interferon-gamma primes macrophages for pathogen ligand-induced killing via a caspase-8 and mitochondrial cell death pathway. Immunity.

[5] Udawatte DJ, Rothman AL (2021). Viral suppression of RIPK1-mediated signaling. mBio.

[6] Chan FK, Luz NF, Moriwaki K (2015). Programmed necrosis in the cross talk of cell death and inflammation. Annu Rev Immunol.

[7] Broz P, Pelegrin P, Shao F (2020). The gasdermins, a protein family executing cell death and inflammation. Nat Rev Immunol.

[8] Liu X, Xia S, Zhang Z, Wu H, Lieberman J (2021). Channelling inflammation: gasdermins in physiology and disease. Nat Rev Drug Discov.

[9] Sauer JD, Pereyre S, Archer KA, Burke TP, Hanson B, Lauer P, Portnoy DA (2011). *Listeria monocytogenes* engineered to activate the Nlrc4 inflammasome are severely attenuated and are poor inducers of protective immunity. Proc Natl Acad Sci U S A.

[10] Warren SE, Duong H, Mao DP, Armstrong A, Rajan J, Miao EA, Aderem A (2011). Generation of a Listeria vaccine strain by enhanced caspase-1 activation. Eur J Immunol.

[11] Cai Z, Jitkaew S, Zhao J, Chiang HC, Choksi S, Liu J, Ward Y, Wu LG, Liu ZG (2014). Plasma membrane translocation of trimerized MLKL protein is required for TNF-induced necroptosis. Nat Cell Biol.

[12] Cho YS, Challa S, Moquin D, Genga R, Ray TD, Guildford M, Chan FK (2009). Phosphorylation-driven assembly of the RIP1-RIP3 complex regulates programmed necrosis and virus-induced inflammation. Cell.

[13] Basil MC, Levy BD (2016). Specialized pro-resolving mediators: endogenous regulators of infection and inflammation. Nat Rev Immunol.

[14] Lamkanfi M, Dixit VM (2010). Manipulation of host cell death pathways during microbial infections. Cell Host Microbe.

[15] Lee S, Karki R, Wang Y, Nguyen LN, Kalathur RC, Kanneganti TD (2021). AIM2 forms a complex with pyrin and ZBP1 to drive PANoptosis and host defence. Nature.

[16] Dai X, Deng Z, Liang Y, Chen L, Jiang W, Zhao W (2020). Enterococcus faecalis induces necroptosis in human osteoblastic MG63 cells through the RIPK3/MLKL signalling pathway. Int Endod J.

[17] Zhang T, Yin C, Boyd DF, Quarato G, Ingram JP, Shubina M, Ragan KB, Ishizuka T, Crawford JC, Tummers B (2020). Influenza virus Z-RNAs induce ZBP1-mediated necroptosis. Cell.

[18] Daniels BP, Kofman SB, Smith JR, Norris GT, Snyder AG, Kolb JP, Gao X, Locasale JW, Martinez J, Gale M (2019). The nucleotide sensor ZBP1 and kinase RIPK3 induce the enzyme IRG1 to promote an antiviral metabolic state in neurons. Immunity.

[19] Yuan F, Cai J, Wu J, Tang Y, Zhao K, Liang F, Li F, Yang X, He Z, Billiar TR (2022). Z-DNA binding protein 1 promotes heatstroke-induced cell death. Science.

[20] Jiao H, Wachsmuth L, Kumari S, Schwarzer R, Lin J, Eren RO, Fisher A, Lane R, Young GR, Kassiotis G (2020). Z-nucleic-acid sensing triggers ZBP1-dependent necroptosis and inflammation. Nature.

[21] Devos M, Tanghe G, Gilbert B, Dierick E, Verheirstraeten M, Nemegeer J, de Reuver R, Lefebvre S, De Munck J, Rehwinkel J, et al. Sensing of endogenous nucleic acids by ZBP1 induces keratinocyte necroptosis and skin inflammation. J Exp Med 2020;217:e20191913. 10.1084/jem.20191913 PMC7336309 32315377

[22] Yang Y, Wu M, Cao D, Yang C, Jin J, Wu L, Hong X, Li W, Lu L, Li J (2021). ZBP1-MLKL necroptotic signaling potentiates radiation-induced antitumor immunity via intratumoral STING pathway activation. Sci Adv.

[23] Muendlein HI, Connolly WM, Magri Z, Smirnova I, Ilyukha V, Gautam A, Degterev A, Poltorak A (2021). ZBP1 promotes LPS-induced cell death and IL-1beta release via RHIM-mediated interactions with RIPK1. Nat Commun.

[24] Wang R, Li H, Wu J, Cai ZY, Li B, Ni H, Qiu X, Chen H, Liu W, Yang ZH (2020). Gut stem cell necroptosis by genome instability triggers bowel inflammation. Nature.

[25] World Medical A (2013). World Medical Association Declaration of Helsinki: ethical principles for medical research involving human subjects. JAMA.

[26] Huleihel L, Dziki JL, Bartolacci JG, Rausch T, Scarritt ME, Cramer MC, Vorobyov T, LoPresti ST, Swineheart IT, White LJ (2017). Macrophage phenotype in response to ECM bioscaffolds. Semin Immunol.

[27] Wang S, Zhang MJ, Wu ZZ, Zhu SW, Wan SC, Zhang BX, Yang QC, Xiao Y, Chen L, Sun ZJ. GSDME is related to prognosis and response to chemotherapy in oral Cancer. J Dent Res. 2022;101:848–58. 10.1177/00220345211073072 35148659

[28] Jiang Y, Fu P, Liu Y, Wang C, Zhao P, Chu X, Jiang X, Yang W, Wu Y, Wang Y (2020). Near-infrared light-triggered NO release for spinal cord injury repair. Sci Adv.

[29] Chen L, Yang QC, Li YC, Yang LL, Liu JF, Li H, Xiao Y, Bu LL, Zhang WF, Sun ZJ (2020). Targeting CMTM6 suppresses stem cell-like Properties and enhances antitumor immunity in head and neck squamous cell carcinoma. Cancer Immunol Res.

[30] Fan W, Li Y, Sun Q, Tay FR, Fan B (2020). Quaternary ammonium silane, calcium and phosphorus-loaded PLGA submicron particles against *Enterococcus faecalis* infection of teeth: an in vitro and in vivo study. Mater Sci Eng C Mater Biol Appl.

[31] Liu J, Wang J, Ren J, Yang Q, Zhan W, Wang M, Hao L, Yue Y (2021). Inhibition of receptor-interacting protein kinase-3 in the necroptosis pathway attenuates inflammatory bone loss in experimental apical periodontitis in Balb/c mice. Int Endod J.

[32] Nikolic N, Jakovljevic A, Carkic J, Beljic-Ivanovic K, Miletic M, Soldatovic I, Andric M, Ivanovic V, Milasin J (2019). Notch signaling pathway in apical periodontitis: correlation with bone resorption regulators and proinflammatory cytokines. J Endod.

[33] Stern MH, Dreizen S, Mackler BF, Selbst AG, Levy BM (1981). Quantitative analysis of cellular composition of human periapical granuloma. J Endod.

[34] Marton IJ, Kiss C (2000). Protective and destructive immune reactions in apical periodontitis. Oral Microbiol Immunol.

[35] Karki R, Sharma BR, Tuladhar S, Williams EP, Zalduondo L, Samir P, Zheng M, Sundaram B, Banoth B, Malireddi RKS (2021). Synergism of TNF-alpha and IFN-gamma triggers inflammatory cell death, tissue damage, and Mortality in SARS-CoV-2 infection and cytokine shock syndromes. Cell.

[36] Bucsan AN, Veatch A, Singh DK, Akter S, Golden NA, Kirkpatrick M, Threeton B, Moodley C, Ahmed M, Doyle LA, et al. Response to hypoxia and the ensuing dysregulation of inflammation impacts mycobacterium tuberculosis pathogenicity. Am J Respir Crit Care Med 2022;206:94–104. 10.1164/rccm.202112-2747OC PMC9718519 35412961

[37] Sarhan J, Liu BC, Muendlein HI, Li P, Nilson R, Tang AY, Rongvaux A, Bunnell SC, Shao F, Green DR, Poltorak A (2018). Caspase-8 induces cleavage of gasdermin D to elicit pyroptosis during Yersinia infection. Proc Natl Acad Sci U S A.

[38] Robinson N, McComb S, Mulligan R, Dudani R, Krishnan L, Sad S (2012). Type I interferon induces necroptosis in macrophages during infection with Salmonella enterica serovar typhimurium. Nat Immunol.

[39] Liu WZ, He MJ, Long L, Mu DL, Xu MS, Xing X, Zeng X, Liao G, Dan HX, Chen QM (2014). Interferon-gamma and interleukin-4 detected in serum and saliva from patients with oral lichen planus. Int J Oral Sci.

[40] Stutz MD, Allison CC, Ojaimi S, Preston SP, Doerflinger M, Arandjelovic P, Whitehead L, Bader SM, Batey D, Asselin-Labat ML (2021). Macrophage and neutrophil death programs differentially confer resistance to tuberculosis. Immunity.

[41] Wang K, Sun Q, Zhong X, Zeng M, Zeng H, Shi X, Li Z, Wang Y, Zhao Q, Shao F, Ding J (2020). Structural mechanism for GSDMD targeting by autoprocessed caspases in pyroptosis. Cell.

[42] Lu Z, Van Eeckhoutte HP, Liu G, Nair PM, Jones B, Gillis CM, Nalkurthi BC, Verhamme F, Buyle-Huybrecht T, Vandenabeele P (2021). Necroptosis signaling promotes inflammation, airway remodeling, and emphysema in chronic obstructive pulmonary disease. Am J Respir Crit Care Med.

[43] Ding J, Wang K, Liu W, She Y, Sun Q, Shi J, Sun H, Wang DC, Shao F (2016). Pore-forming activity and structural autoinhibition of the gasdermin family. Nature.

[44] Zhou B, Abbott DW (2021). Gasdermin E permits interleukin-1 beta release in distinct sublytic and pyroptotic phases. Cell Rep.

[45] Wang C, Yang T, Xiao J, Xu C, Alippe Y, Sun K, Kanneganti TD, Monahan JB, Abu-Amer Y, Lieberman J, Mbalaviele G (2021). NLRP3 inflammasome activation triggers gasdermin D-independent inflammation. Sci Immunol.

[46] Wang Y, Gao W, Shi X, Ding J, Liu W, He H, Wang K, Shao F (2017). Chemotherapy drugs induce pyroptosis through caspase-3 cleavage of a gasdermin. Nature.

[47] Wang X, Jiang W, Yan Y, Gong T, Han J, Tian Z, Zhou R (2014). RNA viruses promote activation of the NLRP3 inflammasome through a RIP1-RIP3-DRP1 signaling pathway. Nat Immunol.

[48] Rebsamen M, Heinz LX, Meylan E, Michallet MC, Schroder K, Hofmann K, Vazquez J, Benedict CA, Tschopp J (2009). DAI/ZBP1 recruits RIP1 and RIP3 through RIP homotypic interaction motifs to activate NF-kappaB. EMBO Rep.

[49] Fernandes-Alnemri T, Yu JW, Datta P, Wu J, Alnemri ES (2009). AIM2 activates the inflammasome and cell death in response to cytoplasmic DNA. Nature.

[50] Shubina M, Tummers B, Boyd DF, Zhang T, Yin C, Gautam A, Guo XJ, Rodriguez DA, Kaiser WJ, Vogel P, et al. Necroptosis restricts influenza a virus as a stand-alone cell death mechanism. J Exp Med 2020;217:e20191259. 10.1084/jem.20191259 PMC7596817 32797196

[51] Kuriakose T, Man SM, Malireddi RK, Karki R, Kesavardhana S, Place DE, Neale G, Vogel P, Kanneganti TD (2016). ZBP1/DAI is an innate sensor of influenza virus triggering the NLRP3 inflammasome and programmed cell death pathways. Sci Immunol.

[52] Shi J, Zhao Y, Wang K, Shi X, Wang Y, Huang H, Zhuang Y, Cai T, Wang F, Shao F (2015). Cleavage of GSDMD by inflammatory caspases determines pyroptotic cell death. Nature.

[53] Maelfait J, Liverpool L, Bridgeman A, Ragan KB, Upton JW, Rehwinkel J (2017). Sensing of viral and endogenous RNA by ZBP1/DAI induces necroptosis. EMBO J.

