# Supplementary material for: Fusobacterium nucleatum triggers proinflammatory cell death via Z-DNA binding protein 1 in apical periodontitis
Source: Cell Commun Signal. 2022 Dec 20;20:196. doi: 10.1186/s12964-022-01005-z (PMC9764563; doi:10.1186/s12964-022-01005-z)
Supplement: Supplementary file 2 — Additional file 1. Table S1. qRT-PCR primers used in the present study. Table S2. siRNA sequences used in the present study. Fig. S1. M0 macrophage differentiated into M1 and M2 phenotype. A mRNA levels of Tnf, Nos2, Il6 and Cd86 in RAW264.7 cells treated with LPS (100 ng/ml) for 24 h (unpaired t test). B mRNA levels of Tgfb, Arg1, Il10 and Cd206 in RAW264.7 cells treated with IL-4 (20 ng/ml) for 24 h (unpaired t test). Data are expressed as the mean ± SEM (*p < 0.05; **p < 0.01; ***p < 0.001). Fig. S2. Enhanced ZBP1 expression in F. nucleatum infected AP tissues and knockdown efficiency of ZBP1. A Immunohistochemistry staining of ZBP1 in the normal and AP tissues (AP, n = 10; Normal, n = 10; Scale bar, 50 μm). B siRNA knockdown efficiency of ZBP1 in RAW264.7 cells (NC, negative control). Fig. S3. siRNA knockdown efficiency of GSDME, MLKL and RIPK3 in RAW264.7 cells. A siRNA knockdown efficiency of GSDME, MLKL and RIPK3 in RAW264.7 cells (NC, negative control). Fig. S4 Immunofluorescence staining of Z-nucleic acid and ZBP1 in RAW264.7 cells treated with Fn-EVs (5 µg/ml) for 12 h (Scale bar, 20 μm). [file 12964_2022_1005_MOESM2_ESM.docx]

**Supporting information**

***Fusobacterium nucleatum* triggers pro****inflammatory cell death *via* activation of Z-DNA binding protein 1 in apical periodontitis**

Hui Liu ^1^, Yuxuan Liu ^1^, Wei Fan ^1,^ *****, Bing Fan ^1,^ *****

^1^ The State Key Laboratory Breeding Base of Basic Science of Stomatology (Hubei-MOST) and Key Laboratory of Oral Biomedicine Ministry of Education, School and Hospital of Stomatology, Wuhan University

237 Luoyu Road, Wuhan 430079, China.

***Corresponding authors:**

Prof. Wei Fan & Bing Fan

Email: weifan@whu.edu.cn; bingfan@whu.edu.cn

**Supplementary Tables**

**Table S1.** qRT-PCR primers used in the present study

| Primers for human genes | | |
| --- | --- | --- |
| Target | Forward primer | Reverse primer |
| *IL1B* | GCCAGTGAAATGATGGCTTATT | AGGAGCACTTCATCTGTTTAGG |
| *IL6* | CACTGGTCTTTTGGAGTTTGAG | GGACTTTTGTACTCATCTGCAC |
| *TNF* | GAGGCCAAGCCCTGGTATG | CGGGCCGATTGATCTCAGC |
| *CXCL10* | CTCTCTCTAGAACTGTACGCTG | ATTCAGACATCTCTTCTCACCC |
| *GAPDH* | CTGGGCTACACTGAGCACC | AAGTGGTCGTTGAGGGCAATG |
| Primers for mouse genes | | |
| *Il1b* | TCGCAGCAGCACATCAACAAGAG | AGGTCCACGGGAAAGACACAGG |
| *Il6* | TAGTCCTTCCTACCCCAATTTCC | TTGGTCCTTAGCCACTCCTTC |
| *Tnf* | TATGGCCCAGACCCTCACA | GGAGTAGACAAGGTACAACCCATC |
| *Cxcl10* | TCCCTATGGCCCTCATTCTCA | CCAAGTGCTGCCGTCATTTTC |
| *Gsdme* | GAGAGTCACTCTTCGTTTGGAA | CTGAAGTACCAGGTTGTCCATA |
| *Caspase-3* | GAAACTCTTCATCATTCAGGCC | GCGAGTGAGAATGTGCATAAAT |
| *Mlkl* | AGCAAGAAGTCCCATATTTGGA | GCTGACATCTGAAACGGTATTC |
| *Zbp1* | AAGAGTCCCCTGCGATTATTTG | TCTGGATGGCGTTTGAATTGG |
| *Arg1* | CTCCAAGCCAAAGTCCTTAGAG | GGAGCTGTCATTAGGGACATCA |
| *Cd86* | TCAATGGGACTGCATATCTGCC | GCCAAAATACTACCAGCTCACT |
| *Nos2* | GGAGTGACGGCAAACATGACT | TCGATGCACAACTGGGTGAAC |
| *Tgfb* | TGATACGCCTGAGTGGCTGTCT | CACAAGAGCAGTGAGCGCTGAA |
| *Gapdh* | TGTGTCCGTCGTGGATCTGA | TTGCTGTTGAAGTCGCAGGAG |

**Table S2.** siRNA sequences used in the present study

| siZBP1 1 | sense | GCUCCAACAAGUGCAGCUUTT |
| --- | --- | --- |
|  | antisense | AAGCUGCACUUGUUGGAGCTT |
| siZBP1 2 | sense | GGACAUAGAAAGCUCUCAATT |
|  | antisense | UUGAGAGCUUUCUAUGUCCTT |
| siGSDME | sense | GCUGCAAACUCCAUGUUAUTT |
|  | antisense | AUAACAUGGAGUUUGCAGCTT |
| siMLKL | sense | GGCAAUGAUAAGAUCCUCUTT |
|  | antisense | AGAGGAUCUUAUCAUUGCCTT |
| siRIPK3 | sense | GGUGAAGGCUAUGGUUAAUTT |
|  | antisense | AUUAACCAUAGCCUUCACCTT |
| Negative control | sense | UUCUCCGAACGUGUCACGUTT |
|  | antisense | ACGUAGCACGUUCGGAGAATT |

**Supplementary Figures**


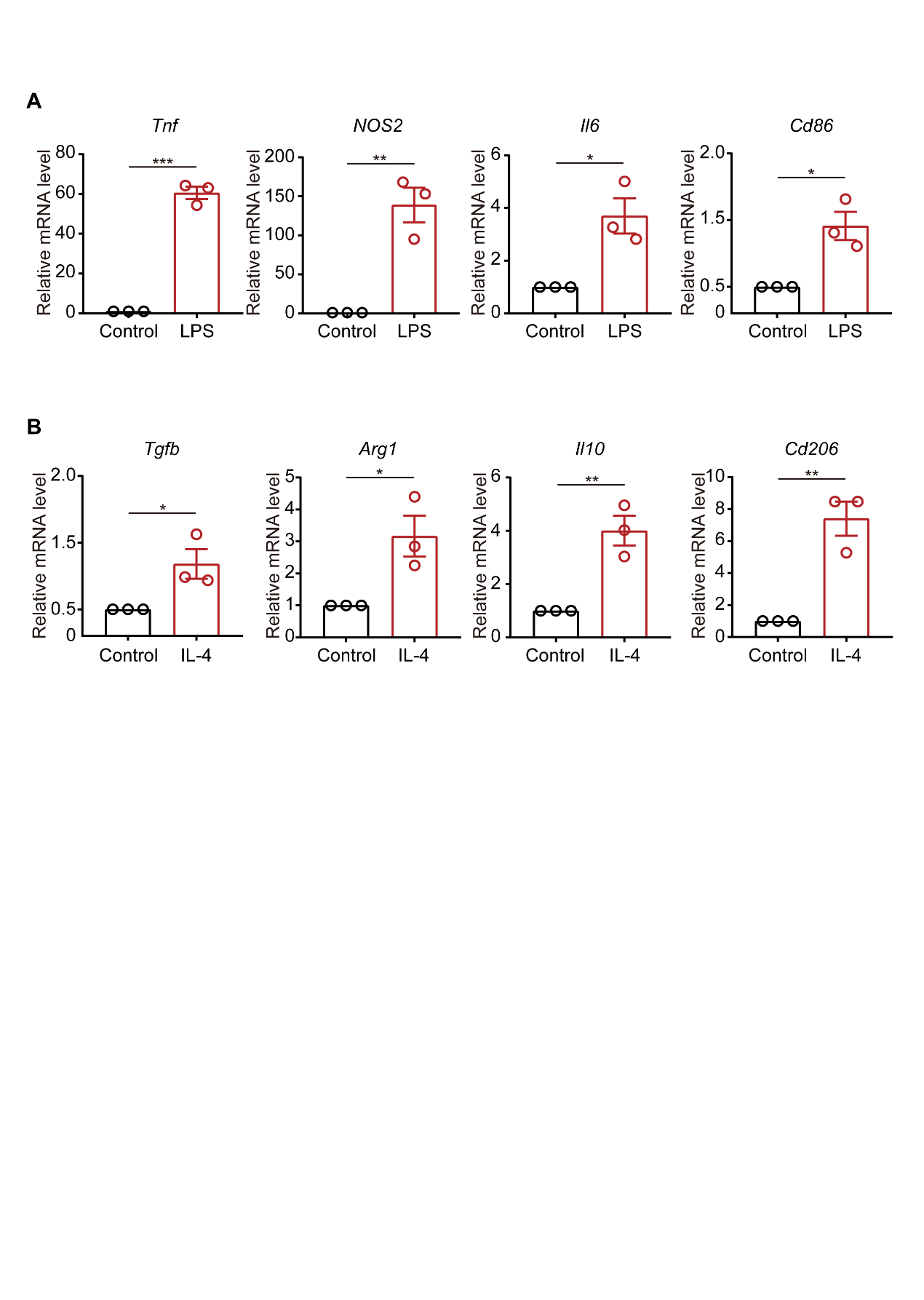


**Figure S1.** M0 macrophage differentiated into M1 and M2 phenotype. **A** mRNA levels of *Tnf*, *Nos2*, *Il6* and *Cd86* in RAW264.7 cells treated with LPS (100 ng/ml) for 24 h (unpaired *t* test). **B** mRNA levels of *Tgfb*, *Arg1*, *Il10* and *Cd206* in RAW264.7 cells treated with IL-4 (20 ng/ml) for 24 h (unpaired *t* test). Data are expressed as the mean ± SEM (*, p < 0.05; **, p < 0.01; ***, p < 0.001).


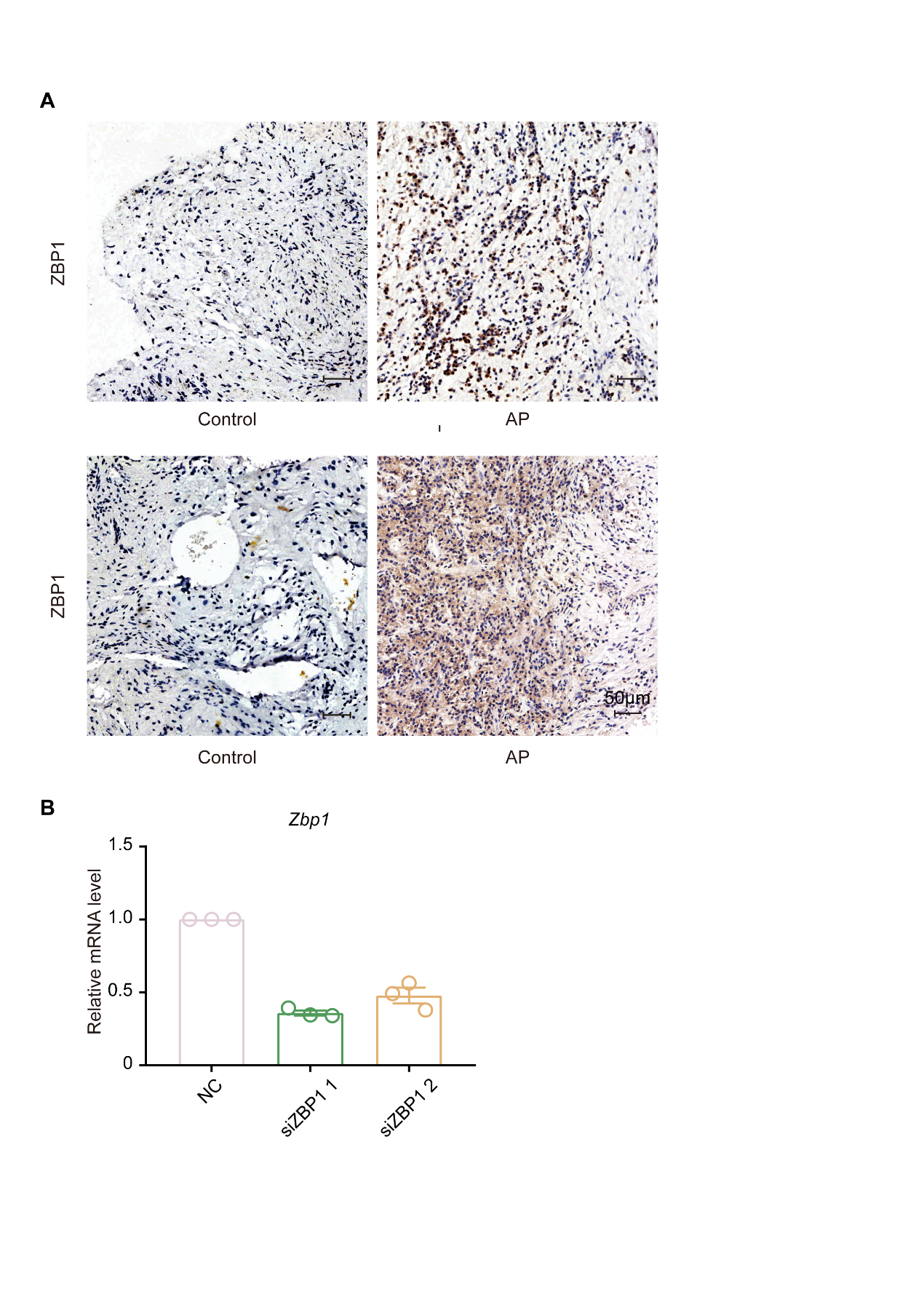


**Figure S2.** Enhanced ZBP1 expression in *F. nucleatum* infected AP tissues and knockdown efficiency of ZBP1. **A** Immunohistochemistry staining of ZBP1 in the normal and AP tissues (AP, n=10; Normal, n=10; Scale bar, 50 μm). **B** siRNA knockdown efficiency of ZBP1 in RAW264.7 cells (NC, negative control).


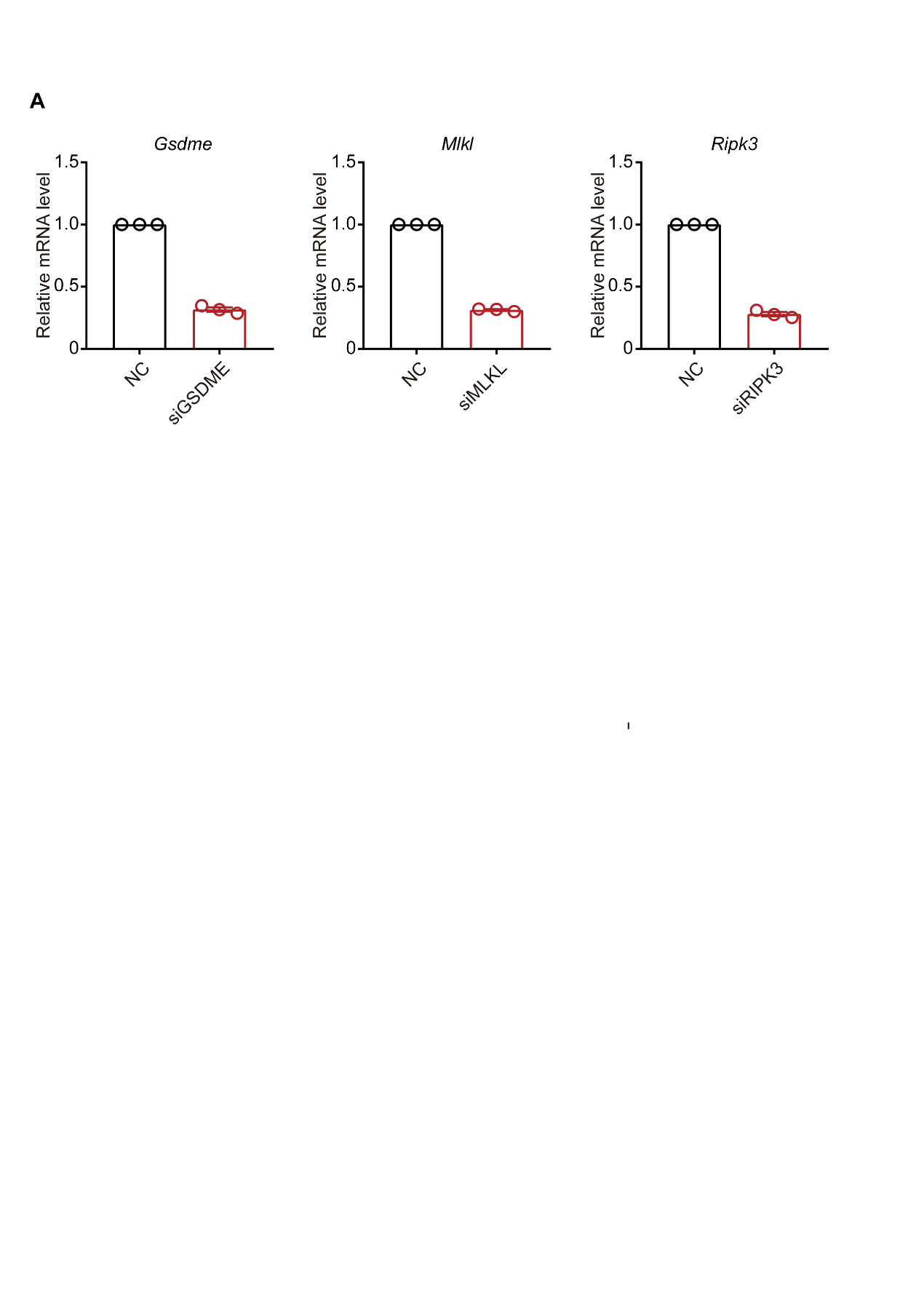


**Figure S3****.** siRNA knockdown efficiency of GSDME, MLKL and RIPK3 in RAW264.7 cells. **A** siRNA knockdown efficiency of GSDME, MLKL and RIPK3 in RAW264.7 cells (NC, negative control).


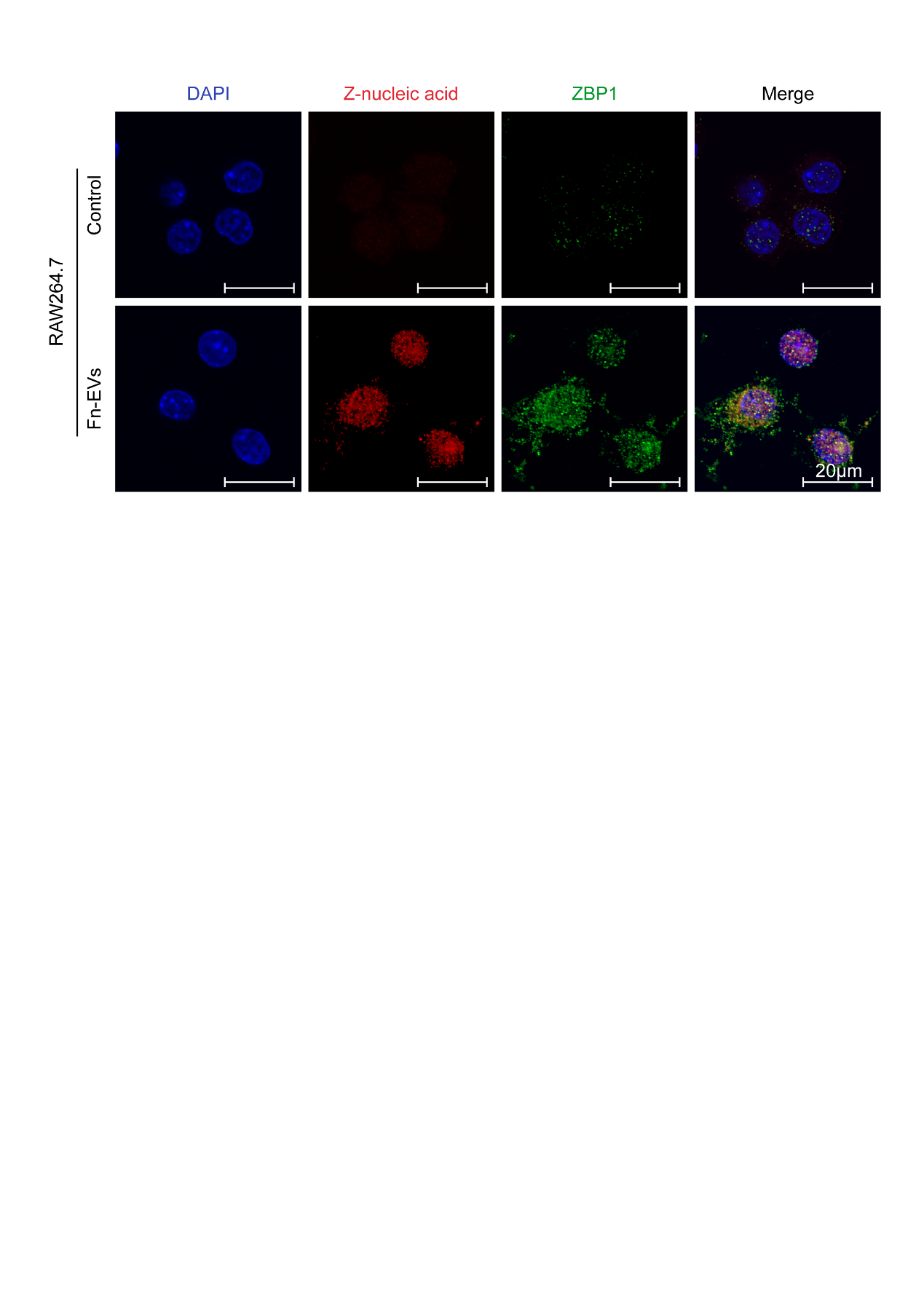


**Figure S4.** Immunofluorescence staining of Z-nucleic acid and ZBP1 in RAW264.7 cells treated with Fn-EVs (5 μg/ml) for 12 h (Scale bar, 20 μm).
